# Supplementary material for: The substrate specificity switch FlhB assembles onto the export gate to regulate type three secretion
Source: Nat Commun. 2020 Mar 10;11:1296. doi: 10.1038/s41467-020-15071-9 (PMC7064499; doi:10.1038/s41467-020-15071-9)
Supplement: Supplementary file 1 — Supplementary Information [file 41467_2020_15071_MOESM1_ESM.pdf]

Supplementary information

The substrate specificity switch FlhB assembles onto the export gate to regulate type three secretion

Lucas Kuhlen et al.

Supplementary Fig. 1 Alignment of FliP sequences.

```
Salmonella_enterica          --------------------MRRLLFLSLAGLWLFSPAAA--------------------        0
Photobacterium_angustum      -----------MLKNKFTLITWMAFLAISVLLSSINIAHA---------VEADVVNSTTA        41
Aliivibrio_fischeri          ---------------MLKTNFIAAVLAAFSLFFMASPAWAVDDLASSIPQTAASAEGMTV        46
Vibrio_mimicus               ------MKRTQRLNLTSWLTTGLLTWLLSGMLGFASLAFAEEPLNTGIPSTAAGASSVTV        55
Vibrio_alginolyticus         ----------MTKGNPIRLLLSTLLLFVGVLFSSFTFAEA---EESALPDNLADGSSVTV        48

Salmonella_enterica          ---------------------AQLPGLISQPLAGGGQSWSLSVQTLVFITSLTFLPAILL        59
Photobacterium_angustum      TAMSAETT---SGHLQAARNGGGIPALSVTVNPDGSEDYSVTLQILALMTALGFLPAMVI        97
Aliivibrio_fischeri          TAMEKEAGAAKTIAKGS-SSGSGIPAFTMTTNANGGEDYSVNLQILGLMTMLGFLPAMVI        104
Vibrio_mimicus               TALEKEQGNAKTIALGSSSGGSGIPAFTMTTNPDGSEDYSINLQILALMTMLGFLPAMVI        114
Vibrio_alginolyticus         QAMQSDTGASRAM---SVSSGGGIPAFTMTTNPDGSEDYSVTLQILALMTMLGFLPAMVI        104
                              :              :   .. :*.:      .*.:.:*:.:* * ::* * ****:::

Salmonella_enterica          MMTSFTRIIIVFGLLRNALGTPSAPPNQVLLGLALFLTFFIMSPVIDKIYVDAYQPFSEQ        119
Photobacterium_angustum      LMTSFTRIVVVMSILRQAMGLQQTPSNQVIIGIALFLTFFVMSPVIDKINANAVQPYINE        157
Aliivibrio_fischeri          LMTSFTRIVVVMSILRQAMGLQQTPSNQVIIGIAMFLTFFIMAPVFDKVNNTAIQPYINE        164
Vibrio_mimicus               LMTSFTRIVVVMSILRQAMGLQQTPSNQVIIGIALFLTFFIMAPVFNQINEQAVQPYLNE        174
Vibrio_alginolyticus         LMTSFTRIVVVMSILRQAMGLQQTPSNQVIIGIALFLTFFVMSPVLNEINDKAVQPYLNE        164
                             :*******::*:.:**:*:*  .:* ***::*:*:*****:*:**::::   * **: ::

Salmonella_enterica          KISMQEALDKGAQPLRAFMLRQTREADLALFARLANSGPLQGPEAVPMRILLPAYVTSEL        179
Photobacterium_angustum      QITAKEALAKAEDPMRQFMLKQTRVKDLETFVNMSGS-TADDPQTVPLTVLIPAFITSEL        216
Aliivibrio_fischeri          EITAREAFDVAQVPMKEFMLAQTRVKDLETFVNMSGV-EAENPEDVPITVVIPAFITSEL        223
Vibrio_mimicus               QISARQAFDLAQEPMKAFMLKQTRIKDLETFVEMSGS-QVTAPEQVSMAVLIPAFITSEL        233
Vibrio_alginolyticus         QVTAREAFDAAQAPMKAFMLKQTRIKDLETFVTMSGE-QVENPEDVSMAVLIPAFITSEL        223
                             ::: ::*:  .  *:: *** ***  **  *. ::.      *: * : :::**::****

Salmonella_enterica          KTAFQIGFTIFIPFLIIDLVIASVLMALGMMMVPPATIALPFKLMLFVLVDGWQLLMGSL        239
Photobacterium_angustum      KTAFQIGFMLFLPFLIIDLVVASILMAMGMMMLSPMIVSLPFKLMLFVLVDGWNLILSTL        276
Aliivibrio_fischeri          KTAFQIGFMLFLPFLIIDLVVASVLMAMGMMMLSPMIVSLPFKLMLFVLVDGWNLILSTL        283
Vibrio_mimicus               KTAFQIGFMLFLPFLIIDLVVASVLMAMGMMMLSPMIVSLPFKLMLFVLVDGWNLILSTL        293
Vibrio_alginolyticus         KTAFQIGFMLFLPFLIIDLVVASVLMAMGMMMLSPMIVSLPFKLMLFVLVDGWNLILSTL        283
                             ******** :*:********:**:***:****: *  ::*************:*::.:*

Salmonella_enterica          AQSFYS       245
Photobacterium_angustum      AGSFGT       282
Aliivibrio_fischeri          AGSFAT       289
Vibrio_mimicus               AGSFAL       299
Vibrio_alginolyticus         AGSFAL       289
                             * **
```

Sequence alignment of FliP sequences from *Salmonella* and various species of the *Vibrionales* order. Red indicates the signal peptide, yellow the N-terminal domain and light blue the part of the sequence that was too poorly ordered to model in the structures of *S.* Typhimurium and *V. mimicus* FliPQR.

Supplementary Fig. 2 Data processing strategy for *V. mimicus* FliPQR

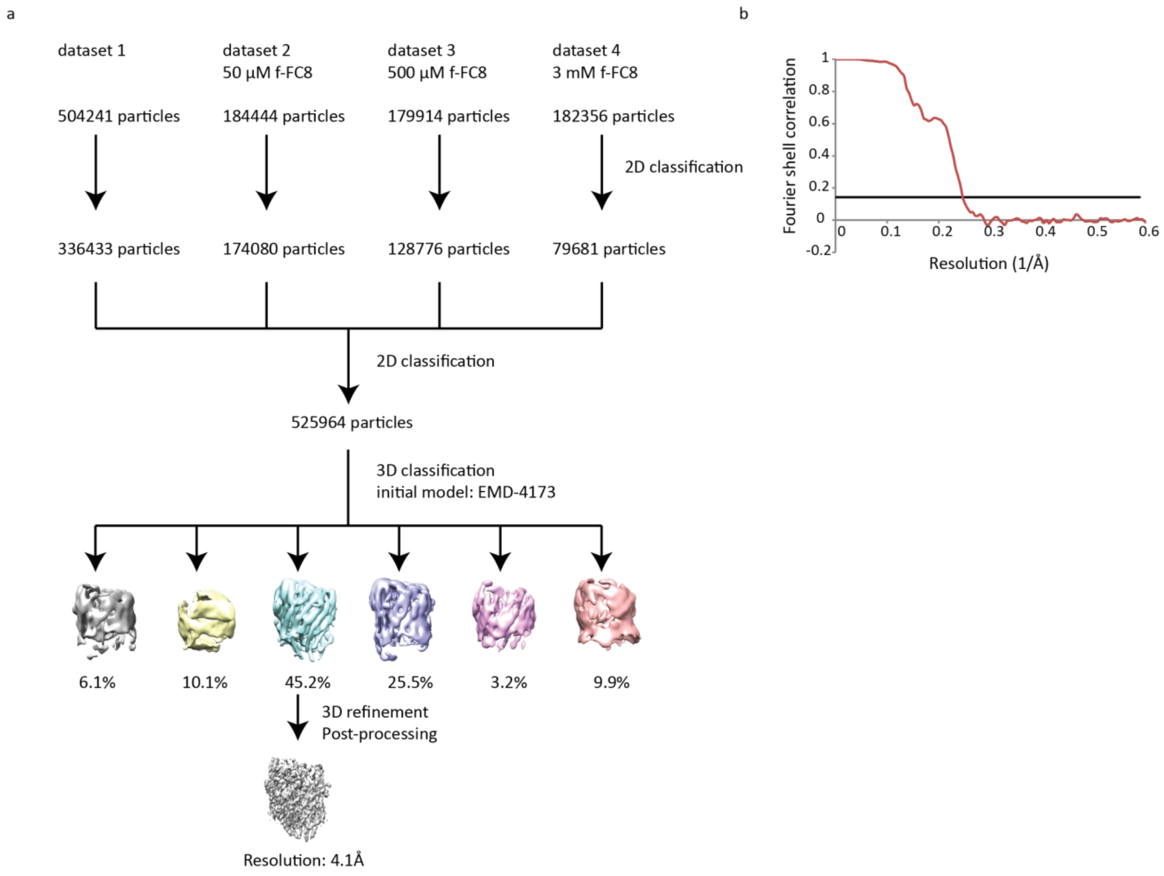

a, Single particle averaging of cryo-EM data of *V. mimicus* FliPQR. **b**, Gold standard FSC curve.

Supplementary Fig. 3 Data processing strategy for *P. savastanoi* FliPQR

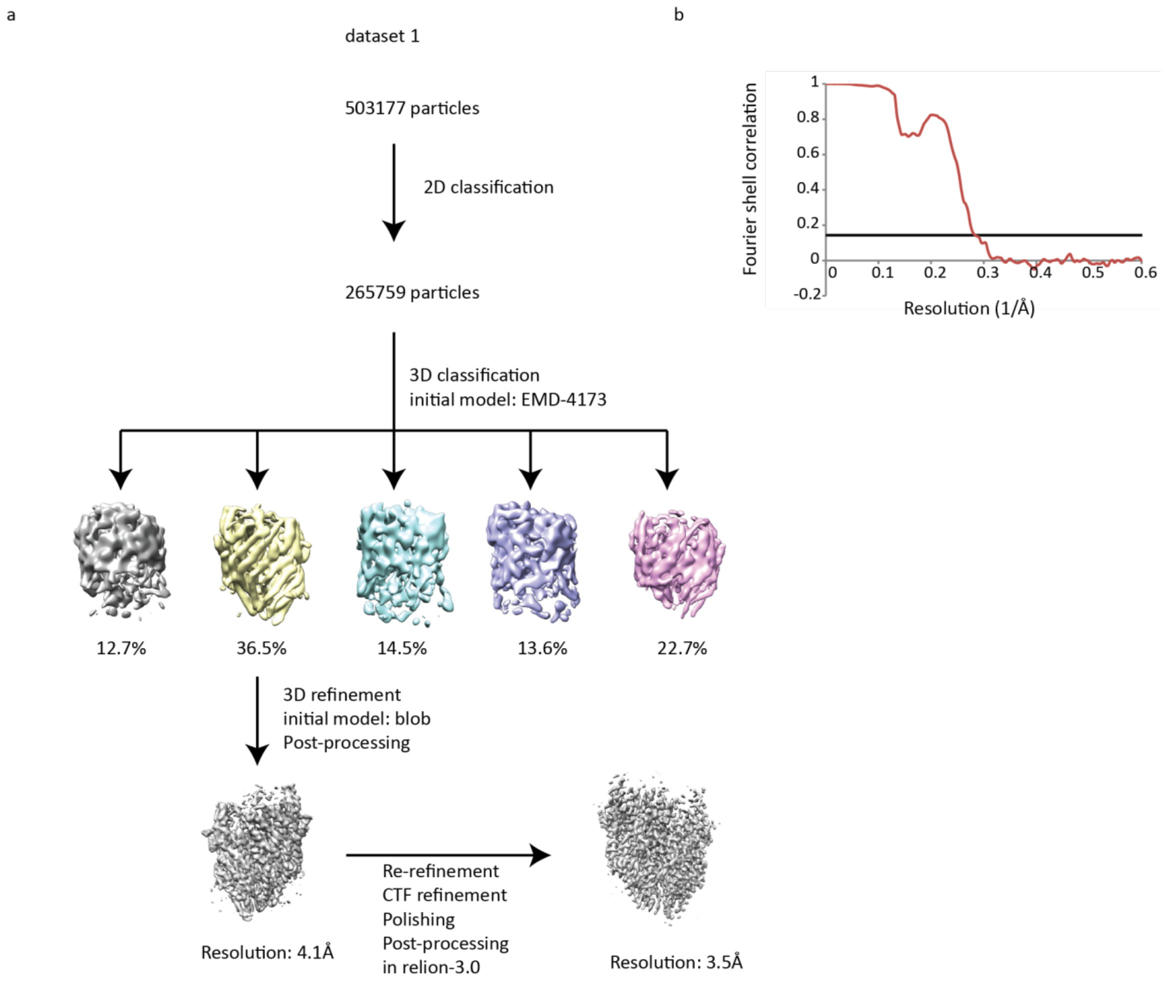

a, Single particle averaging of cryo-EM data of *P. savastanoi* FliPQR. **b**, Gold standard FSC curve.

Supplementary Fig. 4 Mapping the interaction site of SctU by *in vivo* photocrosslinking

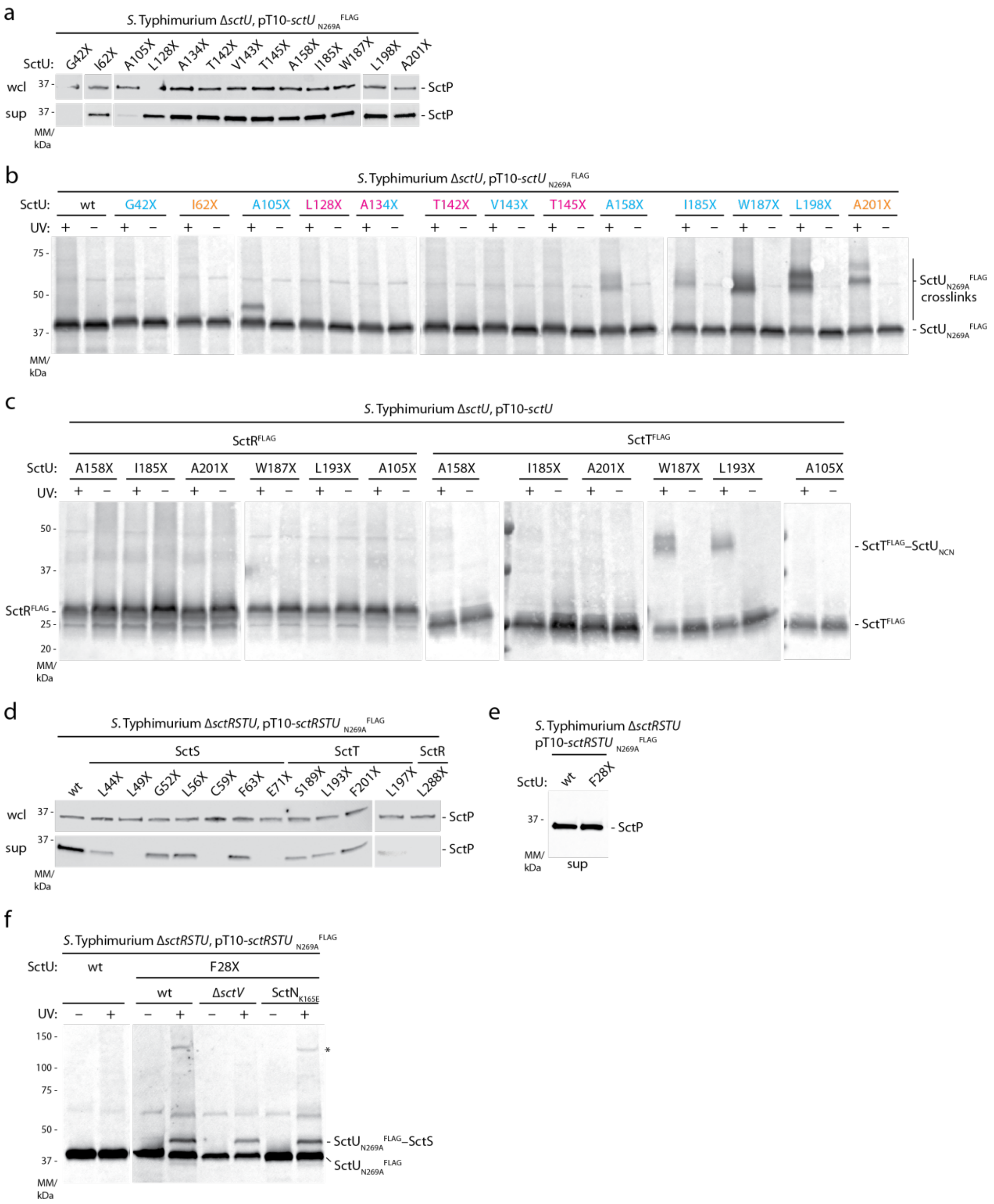

Tested were interaction sites between SctU and SctR, SctS, and SctT, respectively, that where predicted by covariance analysis. **a**, Immunodetection of the early T3SS substrate SctP on Western blots of SDS PAGE-separated whole cell lysates and culture supernatants of the indicated *S*. Typhimurium SctU pBpa mutants. **b**, Immunodetection of $SctU^{FLAG}$ on Western blots of SDS PAGE-separated crude membrane samples of the indicated *S*. Typhimurium SctU pBpa mutants (denoted with X). Each sample is shown with and without UV-irradiation to induce photocrosslinking of pBpa to neighboring interaction partners. Colors indicate covariance predition of SctU to SctR (orange), SctS (magenta), or SctT (cyan). **c**, As in b but showing immunodetection of $SctR^{FLAG}$ or $SctT^{FLAG}$. **d**, As in a but assessing secretion of SctR, SctS, and SctT pBpa mutants, respectively. **e**, Immunodetection of the early T3SS substrate SctP on Western blots of SDS PAGE-separated culture supernatants of the *S*. Typhimurium wild type and a SctU F28pBpa (denoted as X) mutant. **f**, Immunodetection of $SctU^{FLAG}$ on Western blots of SDS PAGE-separated crude membrane samples of *S*. Typhimurium wild type and a SctU F28pBpa mutant. The SctU F28X crosslink was assessed in the wild type, in a ΔsctV and in an ATP hydrolysis-deficient SctN K165E mutant strain. The asterisk indicates the presence of a putative SctU-SctV crosslink.

Supplementary Fig. 5 Data processing strategy for *V. mimicus* FliPQR-FlhB

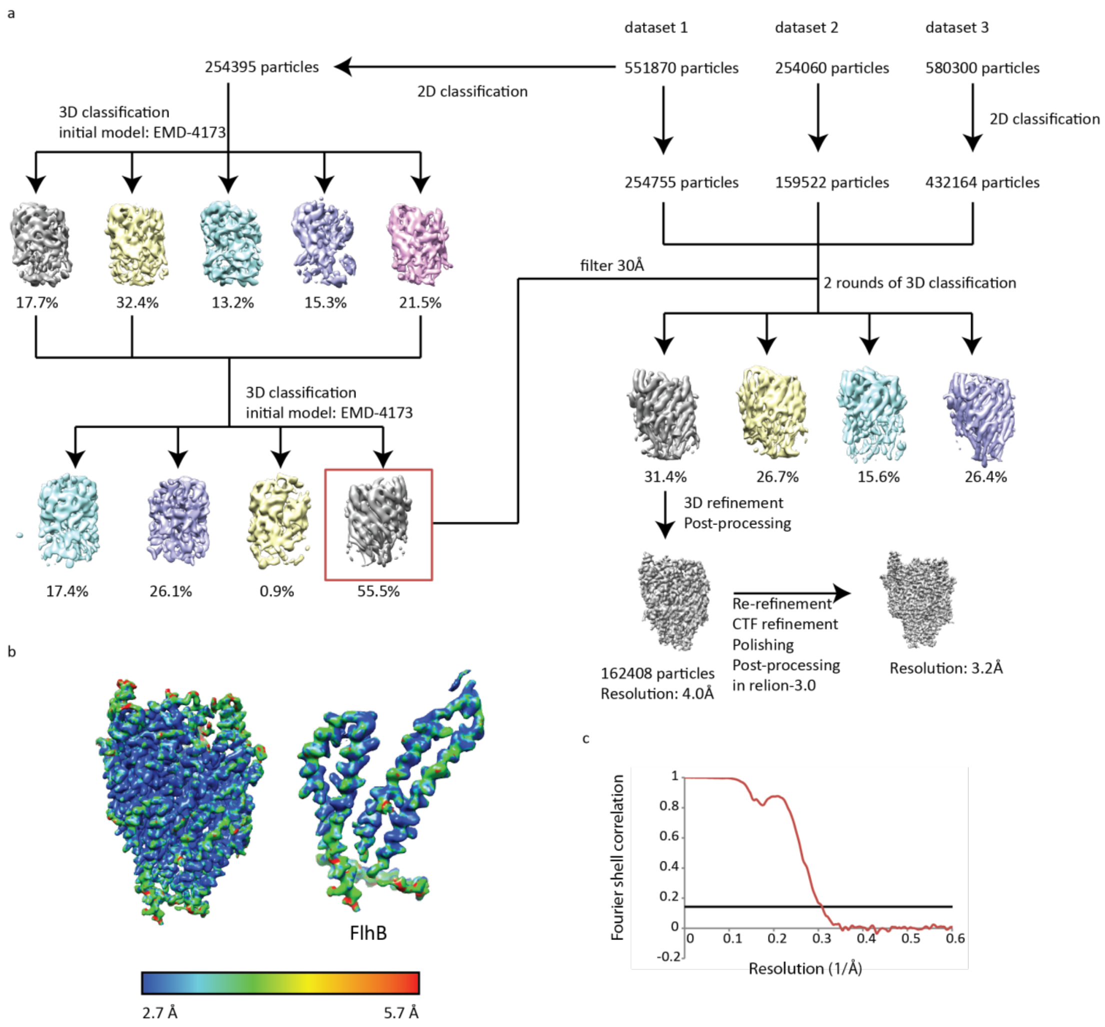

a, Single particle averaging of cryo-EM data of *V. mimicus* FliPQR-FlhB. **b**, Surface representation of the unsharpened volume coloured by local resolution calculated in ResMap. Entire map (left) and zoom of the volume corresponding to FlhB (right). **c**, Gold standard FSC curve.

Supplementary Fig. 6 Reconstruction of FliPQR-FlhB in amphipol

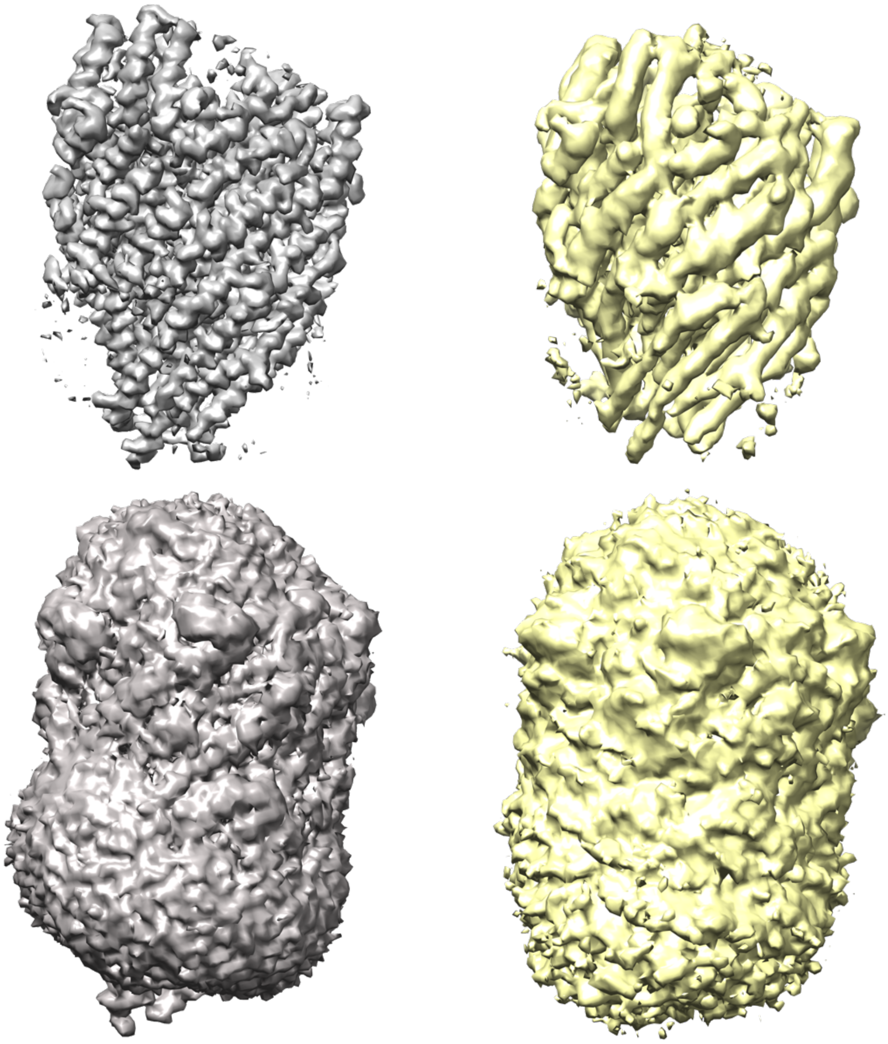

Two contour levels are shown for each volume on LHS (grey) is FliPQRB in LMNG micelle and on RHS (yellow) FliPQRB extracted in DDM and exchanged into amphipol A8-35 for imaging.

Supplementary Fig. 7 Potential lipidic ligands

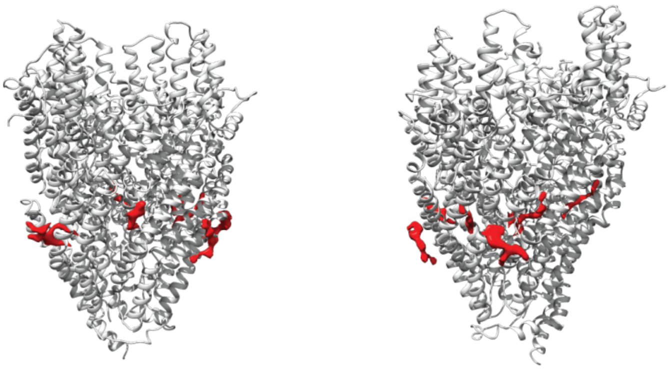

Structure of FliPQR (white) and unmodelled density (red).

Supplementary Fig. 8 Co-variation within FlhB

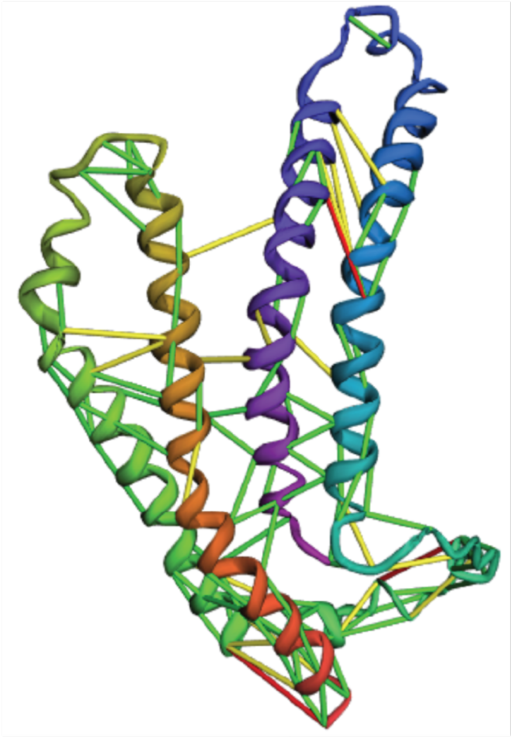

FlhB model with lines connecting co-varying residues. Co-variation was calculated in gremlin and only contacts with a gremlin score higher than 0.75 are displayed. Green lines indicate co-varying residues with a distance < 5 Å, yellow lines 5 – 10 Å and red > 10 Å.

Supplementary Fig. 9 Conservation within the FliPQR-FlhB complex

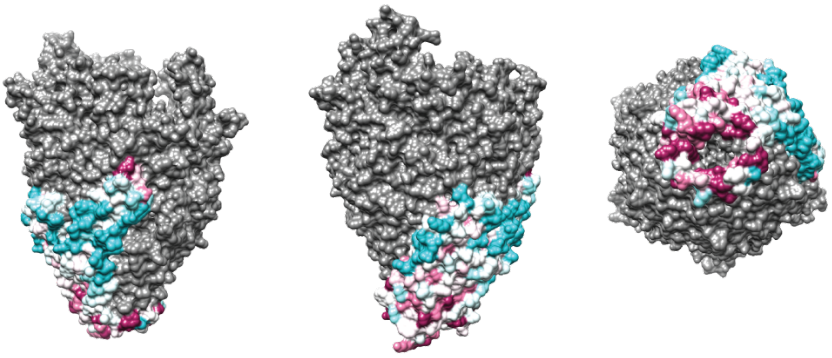

ConSurf representation of sequence conservation of FlhB shows that $FlhB_L$ is highly conserved. Magenta represents highly conserved residues and light blue highly variable residues.

Supplementary Fig. 10 The poorly resolved FlhB N-terminus and $FlhB_{CN}$ are directly underneath the entrance to the lumen of the FliPQR-FlhB complex

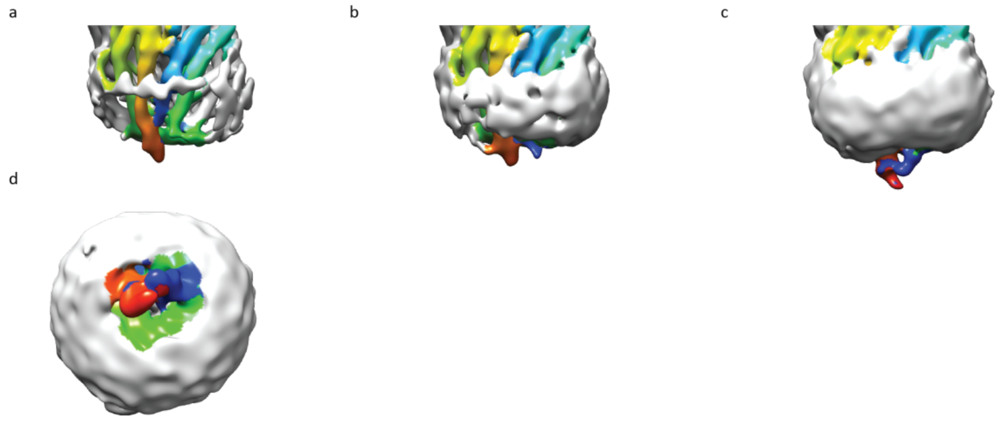

a,b,c, The FliPQR-FlhB cryo-EM density map, filtered to 8 Å, is shown at three different contour levels. Density corresponding to FlhB is coloured in rainbow colours. **d**, View from the cytoplasm shows that the termini of $FlhB_{TM}$ block access to the gate.

Supplementary Fig. 11 Motility of *E. coli* W strains

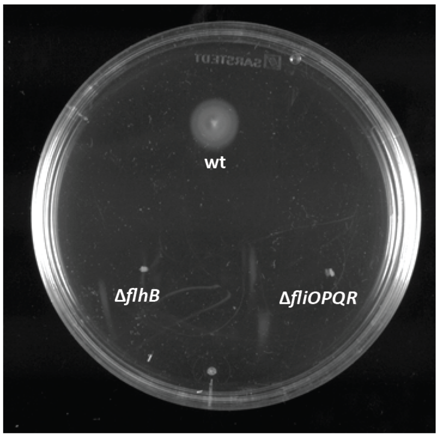

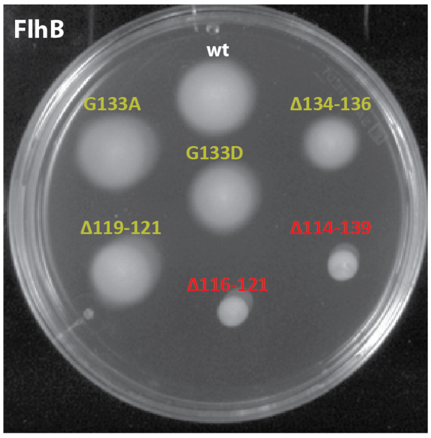

Motility of *E. coli* W wild type, strain WL1 (Δ*fliOPQR*, right) and strain WL2 (Δ*flhB*, left) in soft agar (left) and motility assay of WL2 complemented with *flhB* containing the indicated mutations (right).

Supplementary Fig. 12 Secretion of FliC by *E. coli* W strains

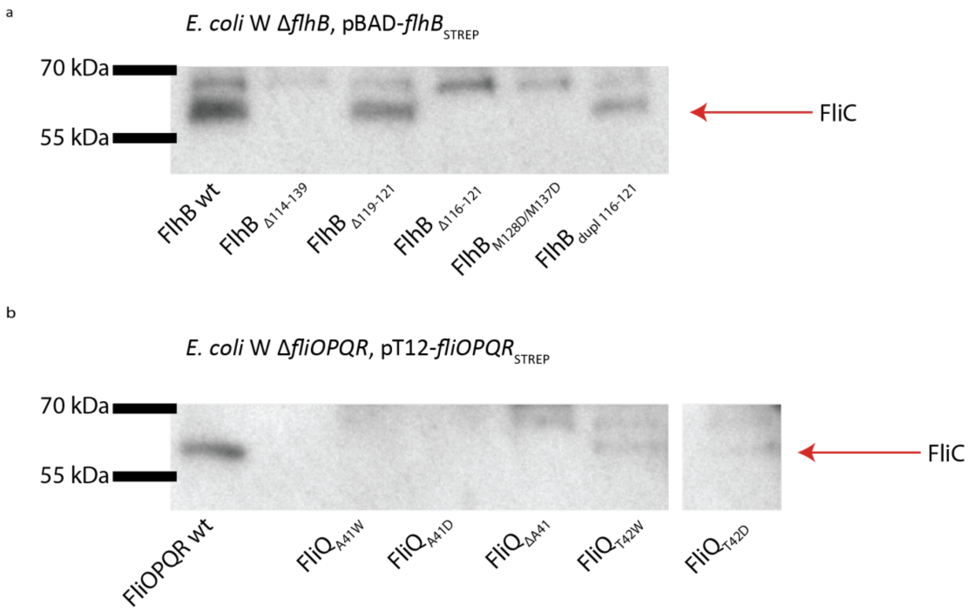

Immunodetection of FliC secreted into the culture media by *E. coli* W Δ*flhB* (**a**) and Δ*fliOPQR* (**b**) complemented with plasmids carrying *flhB* or *fliOPQR* with the indicated mutations.

Supplementary Fig. 13 Presence of flagella on mutant *E. coli* W cells

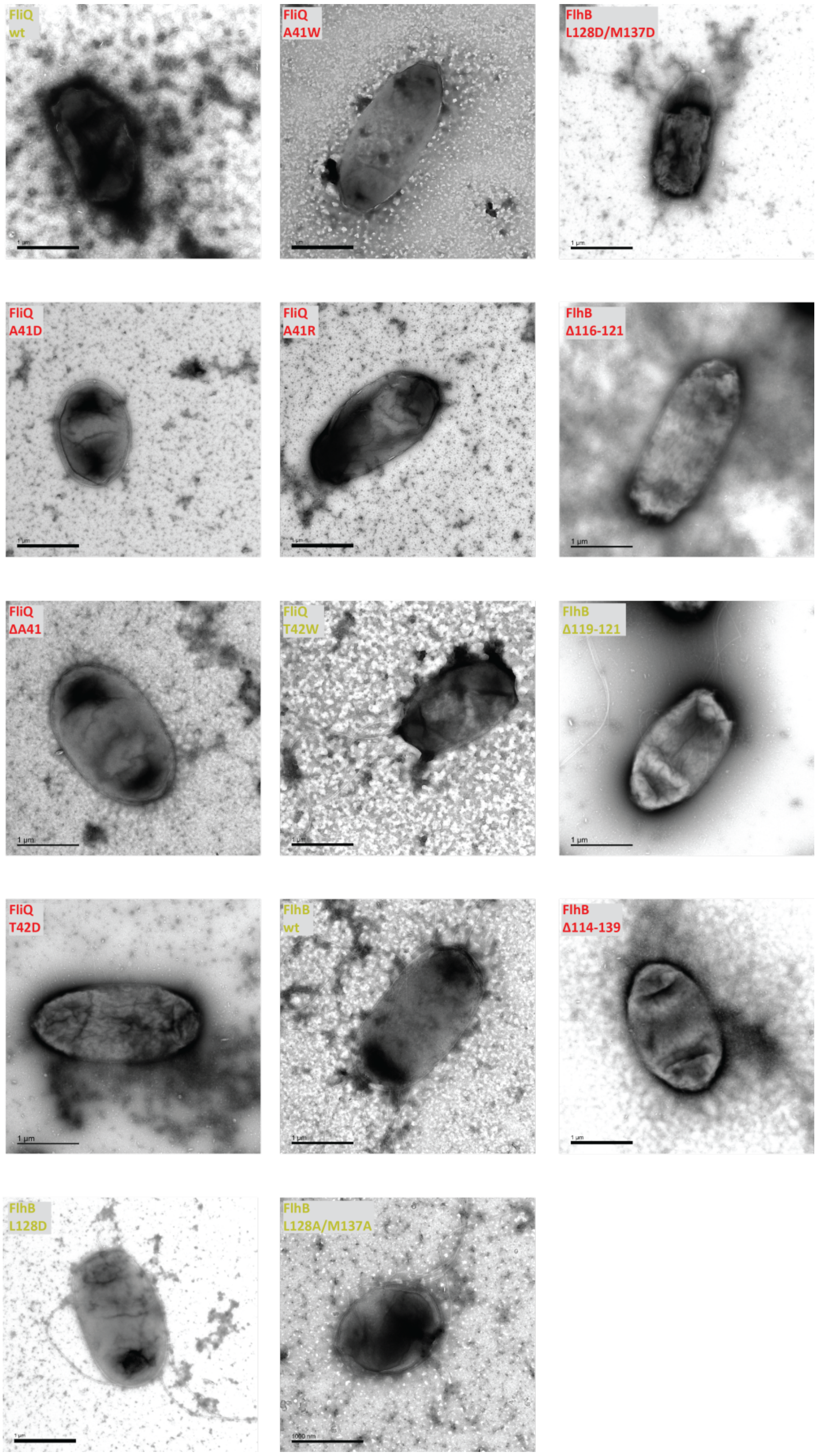

Representative electron microscopy images of *E. coli* W cells showing absence of flagella in non-motile mutants. Scale bar = 1 µm.

Supplementary Fig. 14 Purification of mutant FliPQR FlhB complexes

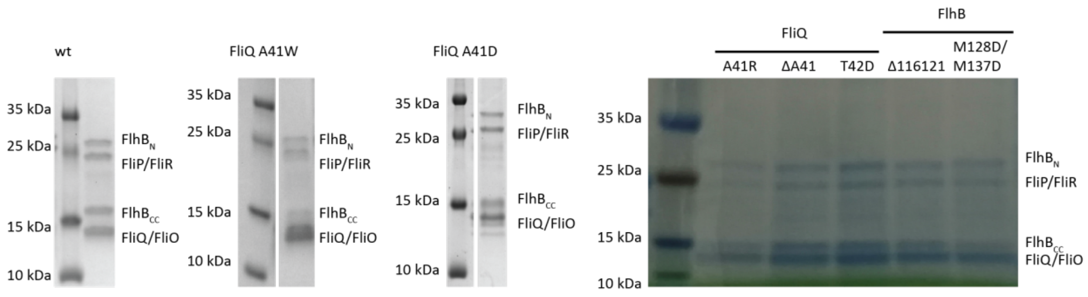

SDS-PAGE analysis of the StrepTrap eluate of FliOPQR-FlhB wild type (left, same as Fig. 2a), FliQ A41W mutant (middle) and FliQ A41D mutant (right).

Supplementary Fig. 15 Photocrosslinking of SctU

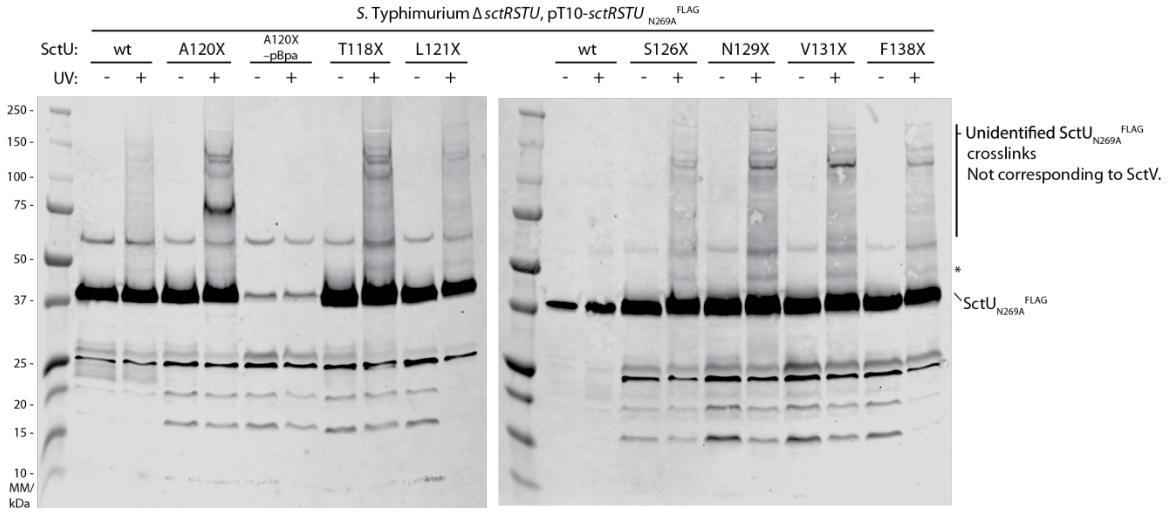

Immunodetection of SctU^{FLAG} on Western blots of SDS PAGE-separated crude membrane samples of the indicated *S.* Typhimurium SctU *p*Bpa mutants (denoted with X). Each sample is shown with and without UV-irradiation to induce photocrosslinking of *p*Bpa to neighboring interaction partners. The – *p*Bpa control shows that little skipping of the amber stop codon occurs in the absence of *p*Bpa. The crosslinks that were specific to the *p*Bpa mutants could not be assigned to SctV or SctS. The asterisk shows a putative SctU-SctS crosslink, which, however, is also slightly visible in one wild type control.

Supplementary tables

Supplementary table 1 – numbering of equivalent residues in different sequences

SctU *S.* Typhimurium	FlhB *V. mimicus*	FlhB *E. coli* W		SctS *S.* Typhimurium	FliQ *V. mimicus*	FliQ *E. coli* W
F24	A28	P27		T38	A40	A40
K27	K31	R30		V39	A41	A41
D28	E32	E31		T40	T42	T42
G38	G42	G41		Q41	S43	Q43
I59	Q62	S61		L42	I44	I44
A94	L105	L104		L47	L49	L49
T107	A118	G117		G50	L52	I52
A109	A120	S119		L54	I56	I56
L110	A121	L120		C57	L59	F59
S115	S126	S125		F61	M63	I63
L117	M128	L127		E69	Q71	N71
N118	N129	N128				
V120	L131	L130		SctT *S.* Typhimurium	FliR *V. mimicus*	FliR *E. coli* W
G122	G133	G132		S186	L189	L188
A123	F134	I133		L190	T193	I192
K125	R136	R135		L194	T197	T196
L126	M137	M136		F198	S201	A200
F127	F138	F137				
T131	S142	T141		SctR *S.* Typhimurium	FliP *V. mimicus*	FliP *E. coli* W
V132	W143	G142		L211	L288	L234
D134	E145	E144				
T135	L146	L145				
K137	K148	K147				
S143	K152	K151				
A149	G158	S157				
I171	H185	N184				
W175	L187	M186				
L182	F193	C192				
A190	L201	V200				
D197	D208	D207				

Supplementary table 2 – primers used

name	number	sequence
pT12_f	1	GAAAACCTGTACTTCCAGGGTC
pT12_r	2	TGGTGAATTCCTCCTGAATTTC
FliO_VIBMI_f	3	TCAGGAGGAATTCACCATGCGAAAATTGGCGGCTCTG
FliR_VIBMI_r	4	GGAAGTACAGGTTTTCACAGTTCAAGCGAATGAGACGACAGATCTGCTC
FlhB_VIBMI_r	5	GGAAGTACAGGTTTTCGTAACGCATATCTGGTGGGATCGGCATATTCTC
FliO_ECOLW_f	6	TTCAGGAGGAATTCACCATGAATAACCACGCTACTGTG
FliR_ECOLW_r	7	TGGAAGTACAGGTTTTCTATTAATGGCAATTCACTAATAATATCAGC
FlhB_ ECOLW_1_f	8	TTCAGGAGGAATTCACCGTGTCTGACGAGAGCGACGACAAAACAG
FlhB_ECOLW_1_r	9	TGGAAGTACAGGTTTTCCTCATGGGTCGGTTTCTCG
FlhB_ECOLW_2_f	10	AACAGGAGGAATTAACCGTGTCTGACGAGAGCGACGACAAAACAG
FlhB_ECOLW 2_r	11	GATGGTCGACGGCGCTTTATTTTTCGAACTGCGGGTGGCTCCACGATCCACCTCCCGATCCACCTCCGGAAC
FliQ ECOLW A41W f	12	TGCAGGCCTGGACGCAGATCAACGAAATGACGCTGTCGTTTATTCC
FliQ ECOLW A41W r	13	ATCTGCGTCCAGGCCTGCAAAATACTGATGATAAGGCCCGTGACCAGCGCCACCAGCAAC
FliQ ECOLW A41D f	14	TGCAGGCCGACACGCAGATCAACGAAATGACGCTGTCGTTTATTCC
FliQ ECOLW A41D r	15	ATCTGCGTGTCGGCCTGCAAAATACTGATGATAAGGCCCGTGACCAGCGCCACCAGCAAC
FliQ ECOLW A41R f	16	TGCAGGCCCGCACGCAGATCAACGAAATGACGCTGTCGTTTATTCC
FliQ ECOLW A41R r	17	ATCTGCGTGCGGGCCTGCAAAATACTGATGATAAGGCCCGTGACCAGCGCCACCAGCAAC
FliQ ECOLW delta A41 f	18	TGCAGGCCACGCAGATCAACGAAATGACGCTGTCGTTTATTCC
FliQ ECOLW delta A41 r	19	ATCTGCGTGGCCTGCAAAATACTGATGATAAGGCCCGTGACCAGCGCCACCAGCAAC
FliQ ECOLW T42W f	20	TGCAGGCCGCCTGGCAGATCAACGAAATGACGCTGTCGTTTATTCC
FliQ ECOLW T42W r	21	ATCTGCCAGGCGGCCTGCAAAATACTGATGATAAGGCCCGTGACCAGCGCCACCAGCAAC
FliQ ECOLW T42D f	22	TGCAGGCCGCCGACCAGATCAACGAAATGACGCTGTCGTTTATTCC
FliQ ECOLW T42D r	23	ATCTGGTCGGCGGCCTGCAAAATACTGATGATAAGGCCCGTGACCAGCGCCACCAGCAAC
FlhB ECOLW L113 deletion r	24	GCCAGAACCGCCTCCCCCTCCCAGCATGACCGGAGAAATGAGCGCCACCAGCAC
FlhB ECOLW S138 deletion f	25	GGAGGCGGTTCTGGCTCGGCTCAGACTGGCGCGGAGTTGCTTAAAGC
pBAD_f	26	AGCGCCGTCGACCATCATC

pBAD_r	27	CATGGTTAATTCCTCCTGTTAGCCC
FlhB ECOLW G123A f	28	CGCTACCGGCCATTAAACGGATGTTCTCGGCTCAGACTG
FlhB ECOLW G123A r	29	CGTTTAATGGCCGGTAGCGGGTTGAGTTTGGAAAACTTCG
FlhB ECOLW G123D f	30	CGCTACCGGACATTAAACGGATGTTCTCGGCTCAGACTG
FlhB ECOLW G123D r	31	CGTTTAATGTCCGGTAGCGGGTTGAGTTTGGAAAACTTCG
FlhB ECOLW I133 deletion f	32	TACCGGGCATGTTCTCGGCTCAGACTGGCGCGGAGTTGCTTAAAGC
FlhB ECOLW R135 deletion r	33	GAGAACATGCCCGGTAGCGGGTTGAGTTTGGAAAACTTCG
FlhB ECOLW Q121 deletion1 f	34	ATTTAGCGGCCAGCCGAAGTTTTCCAAACTCAACCCGCTACCGGGCATTAAACG
FlhB ECOLW G117 deletion r	35	ACTTCGGCTGGCCGCTAAATACCAGCCCTCCCAGCATGACCGGAGAAATGAG
FlhB ECOLW Q121 deletion2 f	36	AGGGCTGGTACAGCCGAAGTTTTCCAAACTCAACCCGCTACCGGGCATTAAACG
FlhB ECOLW V114 deletion r	37	ACTTCGGCTGTACCAGCCCTCCCAGCATGACCGGAGAAATGAGC
FliOPQR KO f	38	CACCGATATCATTACTCCGTCTGAGCGAATGCGTCGCCTGAGCCGTTAGTGATGAATAACGAAGTTCCTATACTTTCTAGAGAATAGGAACTTCggaataggaacttc
FliOPQR KO r	39	TCGCAGGTATTATTTTTCGGATAATCCTTAGGATAACATGATAAACGTTACGGAATTATAGAAGTTCCTATTCTCTAGAAAGTATAGGAACTTCggcgcgcctacctgtg
FlhB KO f1	40	TGTCTGCGAAGTGGTCGATCTTAGCCAGGTAAGC
FlhB KO r1	41	CTGGAAAGAGTATTAAGCGTGAATGATGCCAGAGCGCAAAGC
FlhB KO f2	42	TCATTCACGCTTAATACTCTTTCCAGGATTGGCGACGTGTCTGACGAGAGGAAGTTCCTATACTTTCTAGAGAATAGGAACTTCggaataggaacttc
FlhB KO r2	43	GCATCGCGGCCAGATTACTCATGGGTCGGTTTCTCGTTAATAAAATCCAGGAAGTTCCTATTCTCTAGAAAGTATAGGAACTTCggcgcgcctacctgtg
FlhB KO f3	44	GACCCATGAGTAATCTGGCCGCGATGCTG
FlhB KO r3	45	ACTTTCAATCCAGATAGCATTCAGGCCAAATGC
SpaQ_T38X_QC_f	46	CTGGTAGGGTTATTCCAGTAGGTAACGCAATTACAGGAACAG
SpaQ_T38X_QC_r	47	CTGTTCCTGTAATTGCGTTACCTACTGGAATAACCCTACCAG
SpaQ_V39X_QC_f	48	GGTAGGGTTATTCCAGACGTAGACGCAATTACAGGAACAGACG
SpaQ_V39X_QC_r	49	CGTCTGTTCCTGTAATTGCGTCTACGTCTGGAATAACCCTACC
SpaQ_T40X_QC_f	50	GGGTTATTCCAGACGGTATAGCAATTACAGGAACAGACG
SpaQ_T40X_QC_r	51	CGTCTGTTCCTGTAATTGCTATACCGTCTGGAATAACCC
SpaQ_Q41X_QC_f	52	GTTATTCCAGACGGTAACGTAGTTACAGGAACAGACGCTG

SpaQ_Q41X_QC_r	53	CAGCGTCTGTTCCTGTAACTACGTTACCGTCTGGAATAAC
QC_SpaQ_L42X_f	54	CCAGACGGTAACGCAATAGCAGGAACAGACGCTG
QC_SpaQ_L42X_r	55	CAGCGTCTGTTCCTGCTATTGCGTTACCGTCTGG
SpaS_A94X_QC_f	56	CTGCTCTGCTTAGTGTGTTCCTAGTTACCGGCGTTATTACAGGC
SpaS_A94X_QC_r	57	GCCTGTAATAACGCCGGTAACTAGGAACACACTAAGCAGAGCAG
SpaS_T107X_QC_f	58	CGGTTTTGTGCTGGCGTAGGAAGCATTAAAGCCTAATTTATCGGC
SpaS_T107X_QC_r	59	GCCGATAAATTAGGCTTTAATGCTTCCTACGCCAGCACAAAACCG
SpaS_A109X_QC_f	60	GTTTTGTGCTGGCGACAGAATAGTTAAAGCCTAATTTATCGGCGTTAAACC
SpaS_A109X_QC_r	61	GGTTTAACGCCGATAAATTAGGCTTTAACTATTCTGTCGCCAGCACAAAAC
SpaS_L110X_QC_f	62	CTGGCGACAGAAGCATAGAAGCCTAATTTATCGGCGTTAAAC
SpaS_L110X_QC_r	63	GTTTAACGCCGATAAATTAGGCTTCTATGCTTCTGTCGCCAG
SpaS_S115X_QC_f	64	CAGAAGCATTAAAGCCTAATTTATAGGCGTTAAACCCGGTAG
SpaS_S115X_QC_r	65	CTACCGGGTTTAACGCCTATAAATTAGGCTTTAATGCTTCTG
SpaS_N118X_QC_f	66	GCCTAATTTATCGGCGTTATAGCCGGTAGAAGGGGCAAAAAAAC
SpaS_N118X_QC_r	67	GTTTTTTTGCCCCTTCTACCGGCTATAACGCCGATAAATTAGGC
SpaS_V120X_QC_f	68	CTAATTTATCGGCGTTAAACCCGTAGGAAGGGGCAAAAAAACTTTTTAGTATGC
SpaS_V120X_QC_r	69	GCATACTAAAAAGTTTTTTTGCCCCTTCCTACGGGTTTAACGCCGATAAATTAG
SpaS_F127X_QC_f	70	GGTAGAAGGGGCAAAAAAACTTTAGAGTATGCGCACGGTTAAAG
SpaS_F127X_QC_r	71	CTTTAACCGTGCGCATACTCTAAAGTTTTTTTGCCCCTTCTACC
QC_SpaQ_L47X_f	96	GCAATTACAGGAACAGACGTAGCCTTTTGGCATTAAATTACTTGG
QC_SpaQ_L47X_r	97	CCAAGTAATTTAATGCCAAAAGGCTACGTCTGTTCCTGTAATTGC
QC_SpaQ_G50X_f	98	GGAACAGACGCTGCCTTTTTAGATTAAATTACTTGGCGTGTGTTTATGC
QC_SpaQ_G50X_r	99	GCATAAACACACGCCAAGTAATTTAATCTAAAAAGGCAGCGTCTGTTCC
QC_SpaQ_L54X_f	100	GACGCTGCCTTTTGGCATTAAATTATAGGGCGTGTGTTTATGCTTG
QC_SpaQ_L54X_r	101	CAAGCATAAACACACGCCCTATAATTTAATGCCAAAAGGCAGCGTC
QC_SpaQ_C57X_f	102	GGCATTAAATTACTTGGCGTGTAGTTATGCTTGTTTTTACTGTCTGG

QC_SpaQ_C57X_r	103	CCAGACAGTAAAAACAAGCATAACTACACGCCAAGTAATTTAATGCC
QC_SpaQ_F61X_f	104	GGCGTGTGTTTATGCTTGTAGTTACTGTCTGGCTGGTATG
QC_SpaQ_F61X_r	105	CATACCAGCCAGACAGTAACTACAAGCATAAACACACGCC
QC_SpaQ_E69X_f	106	GTCTGGCTGGTATGGCTAGGTTTTACTCTCTTACGGG
QC_SpaQ_E69X_r	107	CCCGTAAGAGAGTAAAACCTAGCCATACCAGCCAGAC
QC_SpaR_S186X_f	108	GCCTTGGTTCTGGCCTAGCCGGTGGTATTAGTGCTG
QC_SpaR_S186X_r	109	CAGCACTAATACCACCGGCTAGGCCAGAACCAAGGC
QC_SpaR_L190X_f	110	GCCAGTCCGGTGGTATAGGTGCTGTTGCTGTCAG
QC_SpaR_L190X_r	111	CTGACAGCAACAGCACCTATACCACCGGACTGGC
QC_SpaR_F198X_f	112	GCTGTTGCTGTCAGAAGTATAGCTGGGTTTATTGTCGCG
QC_SpaR_F198X_r	113	CGCGACAATAAACCCAGCTATACTTCTGACAGCAACAGC
QC_SpaR_L194X_f	114	GGTGGTATTAGTGCTGTTGTAGTCAGAAGTATTCCTGGG
QC_SpaR_L194X_r	115	CCCAGGAATACTTCTGACTACAACAGCACTAATACCACC
QC_SpaP_L211X_f	116	CTTGATGGCTGGACCTTATAGTCTAAGGGATTGATATTACAGTATATG
QC_SpaP_L211X_r	117	CATATACTGTAATATCAATCCCTTAGACTATAAGGTCCAGCCATCAAG
SpaS_QC_F24X_f	118	CCGCTAAAAAAGGCCAGTCATAGAAAAGTAAAGATCTCATTATCGC
SpaS_QC_F24X_r	119	GCGATAATGAGATCTTTACTTTTCTATGACTGGCCTTTTTTAGCGG
FlhB ECOLW R135A f	120	ATTAAAGCGATGTTCTCGGCTCAGACTGGCGCGGAGTTG
FlhB ECOLW R135A r	121	GAGCCGAGAACATCGCTTTAATGCCCGGTAGCGGGTTGAG
FlhB ECOLW L127A f	122	GTTTTCCAAAGCCAACCCGCTACCGGGCATTAAACGGATGTTCTC
FlhB ECOLW L127A r	123	TTAATGCCCGGTAGCGGGTTGGCTTTGGAAAACTTCGGCTGCAAGGATTTGC
FlhB ECOLW L127D f	124	GTTTTCCAAAGACAACCCGCTACCGGGCATTAAACGGATGTTCTC
FlhB ECOLW L127D r	125	TTAATGCCCGGTAGCGGGTTGTCTTTGGAAAACTTCGGCTGCAAGGATTTGC
FlhB ECOLW M136A f	126	CGCTACCGGGCATTAAACGGGCGTTCTCGGCTCAGACTGGCGCGGAGTTG
FlhB ECOLW M136D f	127	CGCTACCGGGCATTAAACGGGACTTCTCGGCTCAGACTGGCGCGGAGTTG

Supplementary table 3

strains			
name	description	reference	construction note
E. coli W	DSM 1116		
WL1	*E. coli W* ΔFliOPQR	this study	Made by λ Red recombination of the wild type strain with the PCR product of the following primers and template: Primers 38 and 39 and pKD3
WL2	*E. coli W* ΔFlhB	this study	Made by λ Red recombination of the wild type strain with the Gibson assembly product of the PCR products of the following primer/template pairs: Primers 42 and 43 and pKD3, primers 40 and 41 with *E. coli* W genomic DNA and primers 44 and 45 with *E. coli* W genomic DNA.
plasmids			
name	description	reference	construction note
pMIB5689	pT12, $\text{SpaPQR}^{\text{TEV2xStrepII}}$	Dietsche, T., Tesfazgi Mebrhatu, M., Brunner, M. J., Abrusci, P., Yan, J., Franz-Wachtel, M., et al. (2016). Structural and functional characterization of the bacterial type III secretion export apparatus. PLoS Pathogens, 12(12), e1006071. http://doi.org/10.1371/journal.ppat.1006071	
pT12_FliOPQR_PSESH	pT12, *P. savastanoi* $\text{FliOPQR}^{\text{TEV2xStrepII}}$	Kuhlen L., Abrusci P., Johnson S., Gault J., Deme J., Caesar J., Dietsche T., Mebrhatu M. T., Ganief T., Macek B., Wagner S., Robinson C. V., Lea S. M. (2018). Structure of the core of the type III secretion system export apparatus. Nat Struct Mol Biol. 25(7):583-590. https://doi.org/10.1038/s41594-018-0086-9	

pT12_FliOPQR_VIBMI	pT12, *V. mimicus* FliOPQR^{TEV2xStrepII}	this study	Made by Gibson assembly of PCR products of the following two primer/template pairs: 1. Primers 1 and 2 from pMIB5689; 2. primers 3 and 4 from *V. mimicus* genomic DNA.
pT12_FliOPQR_FlhB_VIBMI	pT12, *V. mimicus* FliOPQR FlhB^{TEV2xStrepII}	this study	Made by Gibson assembly of PCR products of the following two primer/template pairs: 1. Primers 1 and 2 from pMIB5689; 2. primers 3 and 5 from *V. mimicus* genomic DNA.
pT12_W1	pT12, *E. coli* W FliOPQR^{TEV2xStrepII}	this study	Made by *in vivo* assembly of PCR products of the following two primer/template pairs: 1. Primers 1 and 2 from pMIB5689; 2. primers 6 and 7 from E. *coli* W genomic DNA.
pT12_W2	pT12, *E. coli* W FliOPQR^{TEV2xStrepII} FliQ A41W	this study	Made by *in vivo* assembly of the PCR product of the following primer/template pair: Primers 12 and 13 from pT12_W1.
pT12_W3	pT12, *E. coli* W FliOPQR^{TEV2xStrepII} FliQ A41D	this study	Made by *in vivo* assembly of the PCR product of the following primer/template pair: Primers 14 and 15 from pT12_W1.
pT12_W4	pT12, *E. coli* W FliOPQR^{TEV2xStrepII} FliQ A41R	this study	Made by *in vivo* assembly of the PCR product of the following primer/template pair: Primers 16 and 17 from pT12_W1.
pT12_W5	pT12, *E. coli* W FliOPQR^{TEV2xStrepII} FliQ ΔA41	this study	Made by *in vivo* assembly of the PCR product of the following primer/template pair: Primers 18 and 19 from pT12_W1.
pT12_W6	pT12, *E. coli* W FliOPQR^{TEV2xStrepII} FliQ T42W	this study	Made by *in vivo* assembly of the PCR product of the following primer/template pair: Primers 20 and 21 from pT12_W1.
pT12_W7	pT12, *E. coli* W FliOPQR^{TEV2xStrepII} FliQ T42D	this study	Made by *in vivo* assembly of the PCR product of the following primer/template pair: Primers 22 and 23 from pT12_W1.
pT12_W8	pT12, *E. coli* W FlhB^{TEV2xStrepII}	this study	Made by *in vivo* assembly of PCR products of the following two

			primer/template pairs: 1. Primers 1 and 2 from pMIB5689; 2. primers 8 and 9 from E. *coli* W genomic DNA.
pBAD_W1	pBAD, *E. coli* W $FlhB^{TEV2xStrepII}$	this study	Made by *in vivo* assembly of PCR products of the following two primer/template pairs: 1. Primers 26 and 27 from pSZ2; 2. primers 10 and 11 from pT12_W8.
pBAD_W2	pBAD, *E. coli* W $FlhB^{TEV2xStrepII}$ G132A	this study	Made by *in vivo* assembly of the PCR product of the following primer/template pair: Primers 28 and 29 from pBAD_W1.
pBAD_W3	pBAD, *E. coli* W $FlhB^{TEV2xStrepII}$ G132D	this study	Made by *in vivo* assembly of the PCR product of the following primer/template pair: Primers 30 and 31 from pBAD_W1.
pBAD_W4	pBAD, *E. coli* W $FlhB^{TEV2xStrepII}$ Δ133-135	this study	Made by *in vivo* assembly of the PCR product of the following primer/template pair: Primers 32 and 33 from pBAD_W1.
pBAD_W5	pBAD, *E. coli* W $FlhB^{TEV2xStrepII}$ Δ118-120	this study	Made by *in vivo* assembly of the PCR product of the following primer/template pair: Primers 34 and 35 from pBAD_W1.
pBAD_W6	pBAD, *E. coli* W $FlhB^{TEV2xStrepII}$ Δ115-120	this study	Made by *in vivo* assembly of the PCR product of the following primer/template pair: Primers 36 and 37 from pBAD_W1.
pBAD_W7	pBAD, *E. coli* W $FlhB^{TEV2xStrepII}$ Δ113-137	this study	Made by *in vivo* assembly of the PCR product of the following primer/template pair: Primers 24 and 25 from pBAD_W1.
pBAD_W8	pBAD, E. coli W FlhBTEV2xStrepII R135A	this study	Made by in vivo assembly of the PCR product of the following primer/template pair: Primers 120 and 121 from pBAD_W1.
pBAD_W9	pBAD, E. coli W FlhBTEV2xStrepII L127A	this study	Made by in vivo assembly of the PCR product of the following primer/template pair: Primers 122 and 123 from pBAD_W1.
pBAD_W10	pBAD, E. coli W FlhBTEV2xStrepII L127D	this study	Made by in vivo assembly of the PCR product of the following primer/template pair: Primers 124 and 125 from pBAD_W1.

pBAD_W11	pBAD, E. coli W FlhBTEV2xStrepII L127A M136A	this study	Made by in vivo assembly of the PCR product of the following primer/template pair: Primers 123 and 126 from pBAD_W1.
pBAD_W12	pBAD, E. coli W FlhBTEV2xStrepII L127D M136D	this study	Made by in vivo assembly of the PCR product of the following primer/template pair: Primers 125 and 127 from pBAD_W1.
pSZ2	pBAD, *S. flexneri* MxiG$^{myc\text{-}His}$	Zenk S. F., Stabat D., Hodgkinson J. L., Veenendaal A. K., Johnson S., Blocker A. J. (2007) Identification of minor inner-membrane components of the Shigella type III secretion system 'needle complex'. Microbiology. 153: 2405–2415. https://doi.org/10.1099/mic.0.2007/007781-0	
pKD46		Datsenko K.A., Wanner B.L. (2000) One-step inactivation of chromosomal genes in Escherichia coli K-12 using PCR products. Proc Natl Acad Sci U S A. 97: 6640-6645. https://doi.org/10.1073/pnas.120163297	
pCP20		Datsenko K.A., Wanner B.L. (2000) One-step inactivation of chromosomal genes in Escherichia coli K-12 using PCR products. Proc Natl Acad Sci U S A. 97: 6640-6645. https://doi.org/10.1073/pnas.120163297	
pKD3		Datsenko K.A., Wanner B.L. (2000) One-step inactivation of chromosomal genes in Escherichia coli K-12 using PCR products. Proc Natl Acad Sci U S A. 97: 6640-6645. https://doi.org/10.1073/pnas.120163297	
pSB3292	pBAD24, HilA	Lara-Tejero, M., Kato, J., Wagner, S., Liu, X., & Galán, J. E. (2011). A sorting platform determines the order of protein secretion in bacterial type III systems. Science (New York, NY), 331(6021), 1188–1191. http://doi.org/10.1126/science.1201476	
pSUP-pBpa		Ryu, Y., & Schultz, P. G. (2006). Efficient incorporation of unnatural amino acids into proteins in Escherichia coli. Nature Methods, 3(4), 263–265. http://doi.org/10.1038/nmeth864	
pMIB6089	pT10, SctRSTU$_{N258A}^{FLAG}$	Dietsche, T., Mebrhatu, M. T., Brunner, M. J., Abrusci, P., Yan, J., Franz-Wachtel, M., ... & Kohlbacher, O. (2016). Structural and functional characterization of the bacterial type III secretion export apparatus. PLoS pathogens, 12(12), e1006071. http://doi:10.1371/journal.ppat.1006071	
pMIB5272	pT10, SctU$_{N258A}^{FLAG}$	Monjarás Feria, J. V., Lefebre, M. D., Stierhof, Y.-D., Galán, J. E., & Wagner, S. (2015). Role of autocleavage in the function of a type III secretion specificity switch protein in *Salmonella enterica* serovar Typhimurium. *mBio*, *6*(5), e01459–15. http://doi.org/10.1128/mBio.01459-15	
pSB3598	pT10, SctU	Wagner, S., Königsmaier, L., Lara-Tejero, M., Lefebre, M., Marlovits, T. C., & Galán, J. E. (2010). Organization and coordinated assembly of the type III secretion export apparatus. Proceedings of the National Academy of Sciences of the United States of America, 107(41), 17745–17750. http://doi.org/10.1073/pnas.1008053107	
pMIB7234	pT10, SctRS$_{T38X}$TU$_{N258A}^{FLAG}$	this study	Made by *in vivo* assembly of the PCR product of the following primer/template pair: Primers 46 and 47 from pMIB6089.
pMIB7235	pT10, SctRS$_{V39X}$TU$_{N258A}^{FLAG}$	this study	Made by *in vivo* assembly of the PCR product of the following primer/template pair: Primers 48 and 49 from pMIB6089.

pMIB7236	pT10, $SctRS_{T40X}TU_{N258A}^{FLAG}$	this study	Made by *in vivo* assembly of the PCR product of the following primer/template pair: Primers 50 and 51 from pMIB6089.
pMIB7237	pT10, $SctRS_{Q41X}TU_{N258A}^{FLAG}$	this study	Made by *in vivo* assembly of the PCR product of the following primer/template pair: Primers 52 and 53 from pMIB6089.
pMIB7027	pT10, $SctRS_{L42X}TU_{N258A}^{FLAG}$	this study	Made by *in vivo* assembly of the PCR product of the following primer/template pair: Primers 54 and 55 from pMIB6089.
pMIB6693	pT10, $SctRSTU_{A94X, N258A}^{FLAG}$	this study	Made by *in vivo* assembly of the PCR product of the following primer/template pair: Primers 56 and 57 from pMIB6089.
pMIB7201	pT10, $SctRSTU_{T107X, N258A}^{FLAG}$	this study	Made by *in vivo* assembly of the PCR product of the following primer/template pair: Primers 58 and 59 from pMIB6089.
pMIB7202	pT10, $SctRSTU_{A109X, N258A}^{FLAG}$	this study	Made by *in vivo* assembly of the PCR product of the following primer/template pair: Primers 60 and 61 from pMIB6089.
pMIB7203	pT10, $SctRSTU_{L110X, N258A}^{FLAG}$	this study	Made by *in vivo* assembly of the PCR product of the following primer/template pair: Primers 62 and 63 from pMIB6089.
pMIB7205	pT10, $SctRSTU_{S115X, N258A}^{FLAG}$	this study	Made by *in vivo* assembly of the PCR product of the following primer/template pair: Primers 64 and 65 from pMIB6089.
pMIB7206	pT10, $SctRSTU_{N118X, N258A}^{FLAG}$	this study	Made by *in vivo* assembly of the PCR product of the following primer/template pair: Primers 66 and 67 from pMIB6089.
pMIB7207	pT10, $SctRSTU_{V120X, N258A}^{FLAG}$	this study	Made by *in vivo* assembly of the PCR product of the following primer/template pair: Primers 68 and 69 from pMIB6089.
pMIB7204	pT10, $SctRSTU_{F127X, N258A}^{FLAG}$	this study	Made by *in vivo* assembly of the PCR product of the following primer/template pair: Primers 70 and 71 from pMIB6089.
pMIB6916	pT10, $SctU_{A149X}$	this study	Made by *in vivo* assembly of the PCR product of the

			following primer/template pair: Primers 86 and 87 from pSB3598.
pMIB6917	pT10, $SctU_{I171X}$	this study	Made by *in vivo* assembly of the PCR product of the following primer/template pair: Primers 88 and 89 from pSB3598.
pMIB7021	pT10, $SctU_{A190X}$	this study	Made by *in vivo* assembly of the PCR product of the following primer/template pair: Primers 94 and 95 from pSB3598.
pMIB6695	pT10, $SctU_{W175X}$	this study	Made by *in vivo* assembly of the PCR product of the following primer/template pair: Primers 90 and 91 from pSB3598.
pMIB6694	pT10, $SctU_{L182X}$	this study	Made by *in vivo* assembly of the PCR product of the following primer/template pair: Primers 92 and 93 from pSB3598.
pMIB6696	pT10, $SctU_{A94X}$	this study	Made by *in vivo* assembly of the PCR product of the following primer/template pair: Primers 56 and 57 from pSB3598.
pMIB7027	pT10, $SctRS_{L42X}TU_{N258A}{}^{FLAG}$	this study	Made by *in vivo* assembly of the PCR product of the following primer/template pair: Primers 54 and 55 from pMIB6089.
pMIB7026	pT10, $SctRS_{L47X}TU_{N258A}{}^{FLAG}$	this study	Made by *in vivo* assembly of the PCR product of the following primer/template pair: Primers 96 and 97 from pMIB6089.
pMIB7023	pT10, $SctRS_{G50X}TU_{N258A}{}^{FLAG}$	this study	Made by *in vivo* assembly of the PCR product of the following primer/template pair: Primers 98 and 99 from pMIB6089.
pMIB7022	pT10, $SctRS_{L54X}TU_{N258A}{}^{FLAG}$	this study	Made by *in vivo* assembly of the PCR product of the following primer/template pair: Primers 100 and 101 from pMIB6089.
pMIB7025	pT10, $SctRS_{C57X}TU_{N258A}{}^{FLAG}$	this study	Made by *in vivo* assembly of the PCR product of the following primer/template pair: Primers 102 and 103 from pMIB6089.
pMIB7032	pT10, $SctRS_{F61X}TU_{N258A}{}^{FLAG}$	this study	Made by *in vivo* assembly of the PCR product of the following primer/template

			pair: Primers 104 and 105 from pMIB6089.
pMIB7031	pT10, $SctRS_{E69X}TU_{N258A}^{FLAG}$	this study	Made by *in vivo* assembly of the PCR product of the following primer/template pair: Primers 106 and 107 from pMIB6089.
pMIB7030	pT10, $SctRST_{S186X}U_{N258A}^{FLAG}$	this study	Made by *in vivo* assembly of the PCR product of the following primer/template pair: Primers 108 and 109 from pMIB6089.
pMIB7029	pT10, $SctRST_{L190X}U_{N258A}^{FLAG}$	this study	Made by *in vivo* assembly of the PCR product of the following primer/template pair: Primers 110 and 111 from pMIB6089.
pMIB7028	pT10, $SctRST_{F198X}U_{N258A}^{FLAG}$	this study	Made by *in vivo* assembly of the PCR product of the following primer/template pair: Primers 112 and 113 from pMIB6089.
pMIB7033	pT10, $SctRST_{L194X}U_{N258A}^{FLAG}$	this study	Made by *in vivo* assembly of the PCR product of the following primer/template pair: Primers 114 and 115 from pMIB6089.
pMIB7024	pT10, $SctR_{L211X}STU_{N258A}^{FLAG}$	this study	Made by *in vivo* assembly of the PCR product of the following primer/template pair: Primers 116 and 117 from pMIB6089.
pMIB7287	pT10, $SctRSTU_{F24X, N258A}^{FLAG}$	this study	Made by *in vivo* assembly of the PCR product of the following primer/template pair: Primers 118 and 119 from pMIB6089.
