# Supplementary material for: The substrate specificity switch FlhB assembles onto the export gate to regulate type three secretion
Source: Nat Commun. 2020 Mar 10;11:1296. doi: 10.1038/s41467-020-15071-9 (PMC7064499; doi:10.1038/s41467-020-15071-9)
Supplement: Supplementary file 3 — Reporting summary [file 41467_2020_15071_MOESM3_ESM.pdf]

nature research

Corresponding author(s): *Double-blind peer review submissions: write DBPR and your manuscript number here instead of author names.*

Last updated by author(s): *YYYY-MM-DD*

Reporting Summary

Nature Research wishes to improve the reproducibility of the work that we publish. This form provides structure for consistency and transparency in reporting. For further information on Nature Research policies, see Authors & Referees and the Editorial Policy Checklist .

Statistics

For all statistical analyses, confirm that the following items are present in the figure legend, table legend, main text, or Methods section.

n/a	Confirmed	
☒	☐	The exact sample size (*n*) for each experimental group/condition, given as a discrete number and unit of measurement
☒	☐	A statement on whether measurements were taken from distinct samples or whether the same sample was measured repeatedly
☒	☐	The statistical test(s) used AND whether they are one- or two-sided<br>*Only common tests should be described solely by name; describe more complex techniques in the Methods section.*
☒	☐	A description of all covariates tested
☒	☐	A description of any assumptions or corrections, such as tests of normality and adjustment for multiple comparisons
☒	☐	A full description of the statistical parameters including central tendency (e.g. means) or other basic estimates (e.g. regression coefficient) AND variation (e.g. standard deviation) or associated estimates of uncertainty (e.g. confidence intervals)
☒	☐	For null hypothesis testing, the test statistic (e.g. *F*, *t*, *r*) with confidence intervals, effect sizes, degrees of freedom and *P* value noted<br>*Give P values as exact values whenever suitable.*
☒	☐	For Bayesian analysis, information on the choice of priors and Markov chain Monte Carlo settings
☒	☐	For hierarchical and complex designs, identification of the appropriate level for tests and full reporting of outcomes
☒	☐	Estimates of effect sizes (e.g. Cohen's *d*, Pearson's *r*), indicating how they were calculated

Our web collection on statistics for biologists contains articles on many of the points above.

Software and code

Policy information about availability of computer code

Data collection	EPU
Data analysis	SIMPLE, RELION 3.0 and 2.0, PHENIX, COOT

For manuscripts utilizing custom algorithms or software that are central to the research but not yet described in published literature, software must be made available to editors/reviewers. We strongly encourage code deposition in a community repository (e.g. GitHub). See the Nature Research guidelines for submitting code & software for further information.

Data

Policy information about availability of data

All manuscripts must include a data availability statement. This statement should provide the following information, where applicable:

- Accession codes, unique identifiers, or web links for publicly available datasets
- A list of figures that have associated raw data
- A description of any restrictions on data availability

Cryo-EM volumes and atomic models have been deposited to the EMDB (accession codes EMD-10095, EMD-10096 and EMD-10093) and PDB (accession codes 6S3R, 6S3S and 6S3L) respectively. The source data underlying Figs 1b-c, 2a,3d and 4b-d and Supplementary Figs 2b, 3b, 4, 5c, 11, 12 and 13 are provided as a Source Data file. Other data are available from the corresponding author upon reasonable request.

Field-specific reporting

Please select the one below that is the best fit for your research. If you are not sure, read the appropriate sections before making your selection.

Life sciences study design

All studies must disclose on these points even when the disclosure is negative.

Sample size	micrographs were collected until sufficient particles were obtained to allow generation of a volume that enabled resolution of secondary structure
Data exclusions	data were excluded at<br>1. micrograph level (based on ctf fit statistics)<br>2. particles level (based on inspection of 2D classes and classification in 3D)
Replication	gold standard FSC methods were used throughout
Randomization	no grouping of samples was appropriate in this case
Blinding	no blinding was performed

Reporting for specific materials, systems and methods

We require information from authors about some types of materials, experimental systems and methods used in many studies. Here, indicate whether each material, system or method listed is relevant to your study. If you are not sure if a list item applies to your research, read the appropriate section before selecting a response.

Materials & experimental systems

n/a	Involved in the study	
☐	☒	Antibodies
☒	☐	Eukaryotic cell lines
☒	☐	Palaeontology
☒	☐	Animals and other organisms
☒	☐	Human research participants
☒	☐	Clinical data

Methods

n/a	Involved in the study	
☒	☐	ChIP-seq
☒	☐	Flow cytometry
☒	☐	MRI-based neuroimaging

Antibodies

Antibodies used	Proteins were detected with primary antibodies anti-St1SctP (InvJ) (1:2000) or M2 anti-FLAG (1:10,000) (Sigma-Aldrich, F3165). Secondary antibodies (Thermo-Fisher, SA5-35571) were goat anti-mouse IgG Dylight 800 conjugate (1:5000).
Validation	commercial antibodies were validated by their manufacturers<br><br>anti SctP<br>17.Monjaras Feria, J.V., Lefebre, M.D., Stierhof, Y.D., Galan, J.E. & Wagner, S. Role of autocleavage in the function of a type III secretion specificity switch protein in Salmonella enterica serovar Typhimurium. MBio 6, e01459-15 (2015).
